# Transactions of the Thirtieth Session of the Homœopathic Medical Society of Pennsylvania

**Published:** 1895-11

**Authors:** 


### TRANSACTIONS OF THE THIRTIETH SESSION OF THE HOMŒOPATHIC MEDICAL SOCIETY OF THE STATE OF PENNSYLVANIA, held at Philadelphia September 18th, 19th, and 20th, 1894. Philadelphia: Sherman & Co., Printers, Seventh and Cherry Streets, 1895.

This volume was issued in March last, and should have been noticed before, but the multiplicity of duties has crowded it into the background.

It is utterly impossible for us to write a criticism of this book that will do it justice. It is, like its predecessors, full of most valuable papers upon the various subjects of surgery, obstetrics, gynæcology, pathology, pædology, materia medica, clinical medicine, ophthalmology, etc. Even the enumeration of these papers is a task that we cannot undertake. The whole collection, it may be stated, however, makes up an elegantly printed volume of nearly four hundred pages. Copies may be obtained of Dr. J. Richey Horner, 79 Arch Street, Allegheny, Pa.

